# Prevalence of high-risk human papillomavirus in oral squamous cell carcinoma with or without chewing habits

**DOI:** 10.1371/journal.pone.0300354

**Published:** 2024-05-01

**Authors:** Namrah Anwar, Qurratulain Chundriger, Sohail Awan, Tariq Moatter, Tazeen Saeed Ali, Maria Abdul Rasheed, Shahid Pervez

**Affiliations:** 1 Department of Pathology and Laboratory Medicine, Aga Khan University Hospital, Karachi, Pakistan; 2 Faculty of Science and Technology, University of Central Punjab, Lahore, Pakistan; 3 Department of Otolaryngology, Head and Neck Surgery, Aga Khan University Hospital, Karachi, Pakistan; 4 School of Nursing and Midwifery, Aga Khan University Hospital, Karachi, Pakistan; University of Vermont College of Medicine: University of Vermont Larner College of Medicine, UNITED STATES

## Abstract

Oral cancer (OC) is the most common cancer in Pakistani males and the second most common in females. Major risk factors include peculiar chewing habits, human papillomavirus (HPV) infection and molecular pathways. However, less data is available for this avertible cancer regarding its association with high-risk HPV (HR-HPV) and chewing habits in this region. Therefore, this study was done to determine the prevalence of HR-HPV in oral squamous cell carcinoma (OSCC) and its correlation with p16 and chewing habits. Formalin-fixed paraffin-embedded (FFPE) biopsy specimens of 186 samples were tested for HR-HPV type 16/18 by PCR, followed by p16 immunostaining (IHC) in a subset of cases (n = 50). Appropriate statistical tests were applied to find the association between HR-HPV/p16 and peculiar chewing habits with significance criteria of p<0.05 with 95% CI. HR-HPV (type 16 &18) was present in seven out of 186 cases (3.8%). Of these seven cases, five were positive for HPV16, whereas two were positive for HPV16/18. The overall expression of p16 protein in 50 samples was 38% (n = 19), and among these 19-IHC positive samples, 26% were positive for HR-HPV DNA. No significant association was found between HR-HPV positivity and p16 and chewing habits (p>0.05). It was concluded that HR-HPV prevalence in OSCC was very low in our population, with no statistically significant correlation with p16 and chewing habits. These results suggest the role of HR-HPV as an independent risk factor in OSCC in the local setting.

## Introduction

The prevalence of oral cancer (OC) statistics vary widely geographically because of different lifestyles, and exposure to various risk factors is more prevalent in developing countries than in the West. The analysis report by GLOBOCAN 2020 presented ~377,713 new cases of lip & oral cavity cancer, which accounts for 2% of all the malignancies globally. Regionally, the most significant proportion of oral cancer is in Asia (65.8%), followed by Europe (17.3%), North America (7.3%), Latin America and the Caribbean (4.7%), Africa (3.8%), and Oceania (1.3%). (gco.iarc.fr). In Asia, the South Asian countries (Bangladesh, Sri Lanka, India, and Pakistan) have the highest mortality and morbidity rates [1].

In Pakistan, according to the most recent figures published by GLOBOCAN 2020, cancer of the lips and oral cavity stands second on the list when both genders combined, with 9.5% new cases in both sexes and 13% the topmost prevalent cancer in males (gco.iarc.fr). According to the latest report of the National Cancer Registry (NCR) from 2015–2019, lip and OC is also the second most common cancer in males and females (8.04%) and has the highest prevalence in males (11.6%). Whereas, in females, it ranks as the third most common cancer (4.85%). Rampant chewing of smokeless tobacco products (SLTs) like betel quid, areca nut, and gutka is one of the main risk factors for high burden of OC in Pakistan [2].

The contributing factors to the progression of Oral squamous cell carcinoma (OSCC) could be biological, like Human papillomavirus (HPV), or molecular signalling transducers. HPV is a non-enveloped virus with 8kb of double-stranded circular DNA and is classified as early genes (E), late genes (L), and viral capsid proteins [3]. It belongs to the Papillomaviridae (PV) family of viruses, divided into 53 genera per recent classification [4]. Based on pathogenic potential, HPV is divided into low-risk HPV and high-risk HPV (HR-HPV). The predominant HR-HPV genotypes associated with HNSCC are 16 and 18 [4]. The course of action of HPV, persistence, clearance, and underlying molecular mechanisms are still under investigation. However, the transformative pathways involving E6 and E7 genes that lead to HPV-induced carcinogenesis are well described. Another oncoprotein, E5, is thought to bind with EGFR early in HPV pathogenesis [4, 5]. High-risk HPV (HR-HPV, type 16 and 18) associated SCC most commonly arises from the oropharynx, primarily the lingual and palatine tonsils. In contrast, the estimated prevalence of HR-HPV in OSCC could range from 6–20% [6].

In general, HPV infects undifferentiated cells in the basal layer of the epithelium and maintains a low copy number while expressing the early genes [7]. Once the viral DNA is integrated into the host cell nucleus, HPV initiates the productive phase of the viral life cycle, and this integration is important for the dysregulation of important mechanisms. For this purpose, the expression of the viral oncoproteins E6 and E7 is crucial. E6 protein induces degradation of p53 through ubiquitin-mediated proteolysis, preventing apoptosis in differentiated and undifferentiated cells. The E7 protein targets and inhibits retinoblastoma (Rb) family members (p105 (RB), p107, and p130), releasing E2F transcription, thus causing proliferation and gene expression [7–9]. p16 is a surrogate marker for HR-HPV prevalence in cervical and oropharyngeal cancers and is overexpressed in transcriptionally active HR-HPV+ve tumors. Under normal circumstances, it acts as a tumor suppressor gene by inhibiting CDK4/6 from retaining the hypophosphorylated status of pRB (remains bound to E2F). In HPV+ve cancers, viral E7 binds to pRB, causing its degradation and bypassing the cell cycle checkpoint. As the following mechanism, p16 expression is stimulated by E2F, resulting in its accumulation in nuclei and cytoplasm [10].

HR-HPV+ and HR-HPV- Head and Neck Squamous Cell Carcinomas (HNSCCs) possess distinct clinical and molecular-genetic characterization with different risk factors. HR-HPV frequency in HNSCC overall is influenced by the study population, site of the tumors, and detection methods [11]. However, with low percentages, HR-HPV positivity is an independent risk factor for the HNSCC, especially OSCC.

We have previously reported a significant association between chewing habits, mostly betel quid, areca nut, and gutka and low SES with OSCC in the local population. Male patients with low SES in their forties who were addicted to chewing for years constituted the bulk of OSCC. Buccal mucosa was the most common site in chewers, and most presented with late-stage tumors [12]. Continuing the study, we aimed to estimate HR-HPV and p16 positivity among OSCC patients and their association with chewing status in a subset of the Pakistani population.

## Methodology

Detailed methodology of study design, sample size, and data collection (demographic characteristics, socioeconomic factors, clinicopathological features and chewing habits) has been published [12]. Briefly, 186 OSCC-diagnosed patients in 2017 were recruited in a cross-sectional study setting. Only those patients (>18 years of age) who were diagnosed and treated for OSCC at Aga Khan University Hospital (AKUH) were recruited

### Ethical approval

Under an Institutional Review Board approval at AKUH, ethical approval for the current study was obtained (via its letter 4091-Pat-ERC, dated June 16, 2016). Patients who gave consent before surgery were recruited for their sample to be used for research purposes. Approval was also taken before the questionnaire, and in the case of the deceased, consent and information were obtained from an immediate family member. Those not fulfilling inclusion criteria or refusing to provide information were excluded from the study.

### Experimental methodology

#### DNA extraction

HR-HPV detection, i.e., HR types 16 and 18, were checked with PCR using the DNA as an input sample. According to the manufacturer’s protocol, total DNA was extracted from OSCC biopsy blocks using the FFPE DNA isolation kit from Qiagen, Cat #56404. However, the protocol was optimized as needed. Briefly, eight sections of 10μm from the FFPE block were collected in 1.5 ml eppendorf tubes. Tubes were kept on a heat block at 65°C to melt the paraffin. One ml of xylene was added to the tubes to dissolve the paraffin. Tubes were vortexed briefly and centrifuged at full speed, i.e., 14000rpm for 2 mins. The step was repeated until the paraffin was dissolved completely. The supernatant was discarded, and 1 ml of 100% ethanol was added to wash off the xylene. Tubes were vortexed and centrifuged at full speed for 2 mins, the supernatant was discarded, and the step was repeated. The tissue was then recovered with one ml of 70% ethanol twice using the same vortex and centrifuge conditions. The supernatant was discarded, and the pellet was air dried. Pellet was resuspended in 180μl Buffer ATL, and 20 μl proteinase K and incubation was done at 56°C for 16h for complete cell lysis. The manufacturer’s protocol was followed for the downstream steps. To elute the DNA, 30μl of elution buffer was added directly to the spin column and incubated at room temperature for 15–20 mins. After centrifugation, the same flow-through was applied again to the column for a better DNA yield. DNA was quantified by measuring 260/280 OD with Nanodrop.

#### Polymerase Chain reaction (PCR)

PCR of isolated genomic DNA was performed to get the desired amplified product of HR-HPV. Previously reported primers for HPV-16/18 [13] and β-Globin as a housekeeping gene were used (Primers sequences in Table 1). For PCR, GoTaq® Flexi DNA Polymerase kit from Promega (Cat#M8295) was used. HPV-16/18 primers were optimized on diagnosed cervical cancer samples. In a final volume of 20μl/reaction, 4μl of 5X Flexi buffer (final concentration 1X), 2μl of 2mM dNTPs mix (0.2mM each dNTP), 3μl MgCl_2,_ 0.5μl each of 10μM forward and reverse primers, 0.1μl of DNA Polymerase, and 2μl of template DNA were added. The PCR conditions were: Initial Denaturation at 95°C for 2 mins (1 cycle), 30 cycles of denaturation at 95°C for 35s, annealing at 56°C for 1 min, extension at 72°C for 35s, followed by one cycle of final extension at 72°C for 7 mins. Positive and negative controls were cervical cancer and non-template sample, respectively and the samples were run in triplicates. Products were separated on 2% Agarose gel electrophoresis using a DNA ladder of 100bp and 50bp. The gel was visualized on Bio-Rad GelDoc, and results were recorded as positive and negative.

**Table 1:** Primer sequences of HPV 16/18 and β -Globin for PCR.

Gene	Primer sequence 5’>3’	Product length (bp)
HPV-16-F	TCAAAAGCCACTGTGTCCTG	120
HPV-16-R	CGTGTTCTTGATGATCTGCA	
HPV-18-F	ACCTTAATGAAAAACGACGA	100
HPV-18-R	CGTCGTTGGAGTCGTTCCTG	
β-globin-F	ACACAACTGTGTTCACTAGC	110
β-globin-R	CAACTTCATCCACGTTCACC	

#### Immunohistochemistry of p16 (INK4A)

In a subset of samples (n = 50), protein expression of p16 was determined by performing IHC using CINtec® p16 antibody E6H4 clone (Ventana, Tucson, AZ, USA) [14] and DAKO visualization system. These 50 samples were chosen randomly through computer-generated numbers. Sections of 3–4μm were taken on slides and dewaxed for 25–30 mins at 65°C. Xylene was used to deparaffinize the tissue, and tissue was recovered with subsequent dips in 100% (for 2 mins) and 70% ethanol (for 3 mins). Slides were then washed with water and dipped in 200μl Retrieval solution (1X) at 97°C for 30–60 mins. Blocking was performed by incubation in H_2_O_2_ for 5 mins. After washing, slides were incubated with primary antibody for 20–25 mins. Slides were again washed and incubated with 50–100μl of secondary antibody (HRP) for 25 mins. DAB chromogen was added (50–100μl), and slides were left for 5 mins. Incubation in DAB, HRP substrate, revealed antigenic localization. Counterstaining with Mayer’s Haematoxylin was done for 5 mins, and tissues were dehydrated in graduated Ethanol (75% and 100% for 2 mins each). After 2 mins incubation in xylene, slides were directly mounted. Histopathological analysis was done for nuclear and cytoplasmic staining using Olympus Light Microscope with a magnification range of 2X to 40X. The cutoff percentage for p16 positivity was set at >10% of cells showing nuclear and cytoplasmic block staining of tumor cells [15]. Results were recorded as positive (focal to diffuse block staining) and negative.

### Statistical analysis

Data were analyzed for association using the SPSS package 22 (IBM, Rochester, USA). HR-HPV status and p16 positivity were checked for significance with chewing habits overall and chewing substances (betel quid, gutka, and areca nut) by logistic regression. Odds ratios and their respective 95% confidence intervals (CI) were estimated using univariate and multivariate logistic regression, keeping this variable as the dependent variable. A cutoff p-value<0.2 was chosen in univariate regression to perform multivariate regression analysis [16]. An insignificant p-value of chi-square in Hosmer and Lemeshow Test validated the goodness-of-fit of the model. The Chi-square test was used to study the correlation between HR-HPV positivity by PCR and IHC p16. All analyses were set as two-sided, and a p<0.05 was considered significant.

## Results

### HR-HPV and p16 status

**Fig 1:**
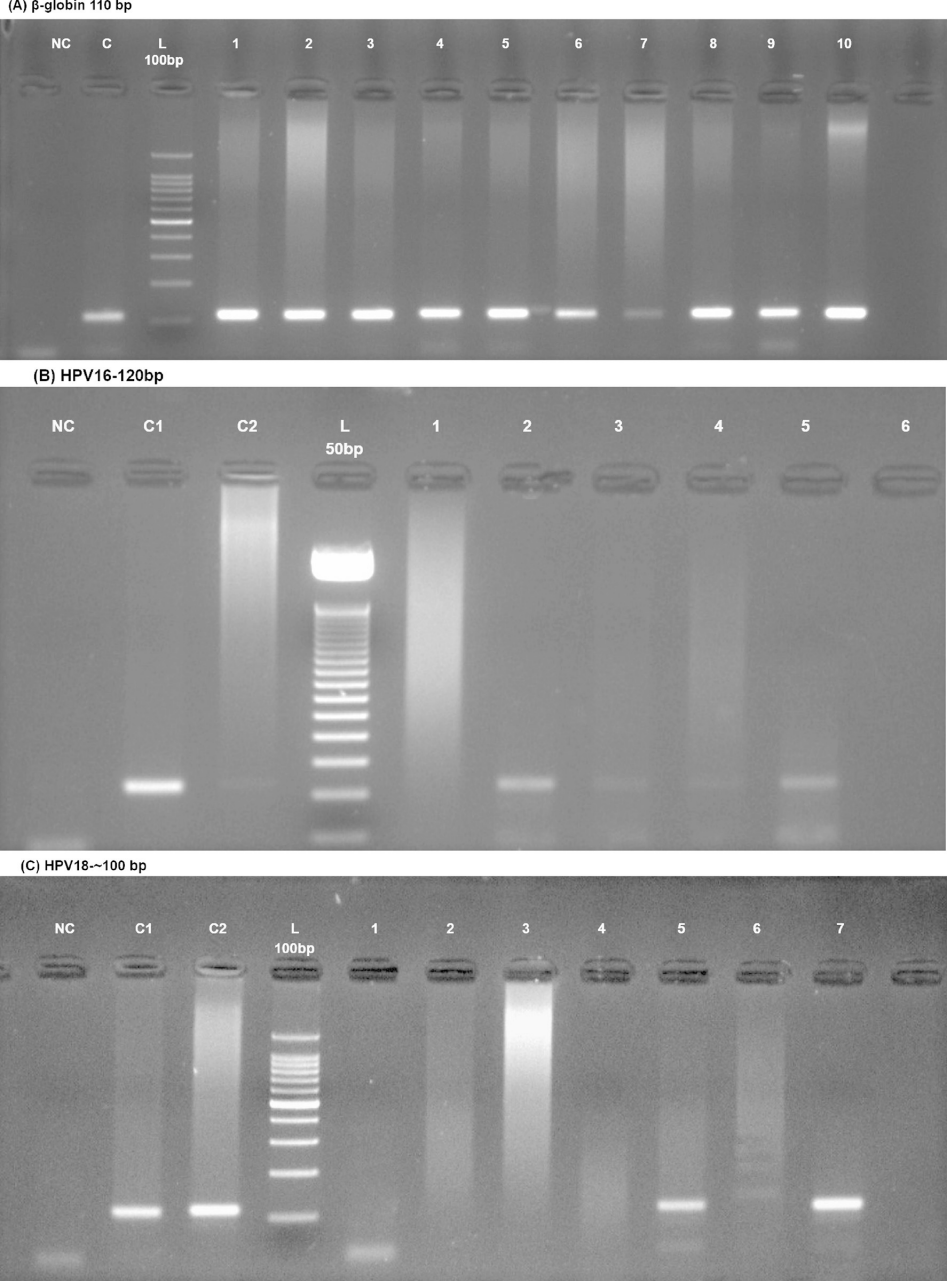
Gel electrophoresis of HPV16/18 and β-globin. (A) shows the presence of β-globin (110bps) in a set of samples. (B) presents the image of HPV-16 (120bp). (C) represents HPV-18 positivity (~100bp). The labelling of gel corresponds to NC = negative control, C = positive control (cervical cancer), C1/C2 positive control (cervical cancer), L = ladder. The numbering of the wells given is random. Well number 2 &5 in (B) are equivalent to 5 & 7 in (C), respectively.

PCR-based HR-HPV detection in all the samples (N = 186) resulted in only 3.8% (n = 7) positive cases (Fig 1A–1C). Among these seven positive samples, two were positive for HPV 16 and 18, while others were positive for only HPV-16.

**Fig 2:**
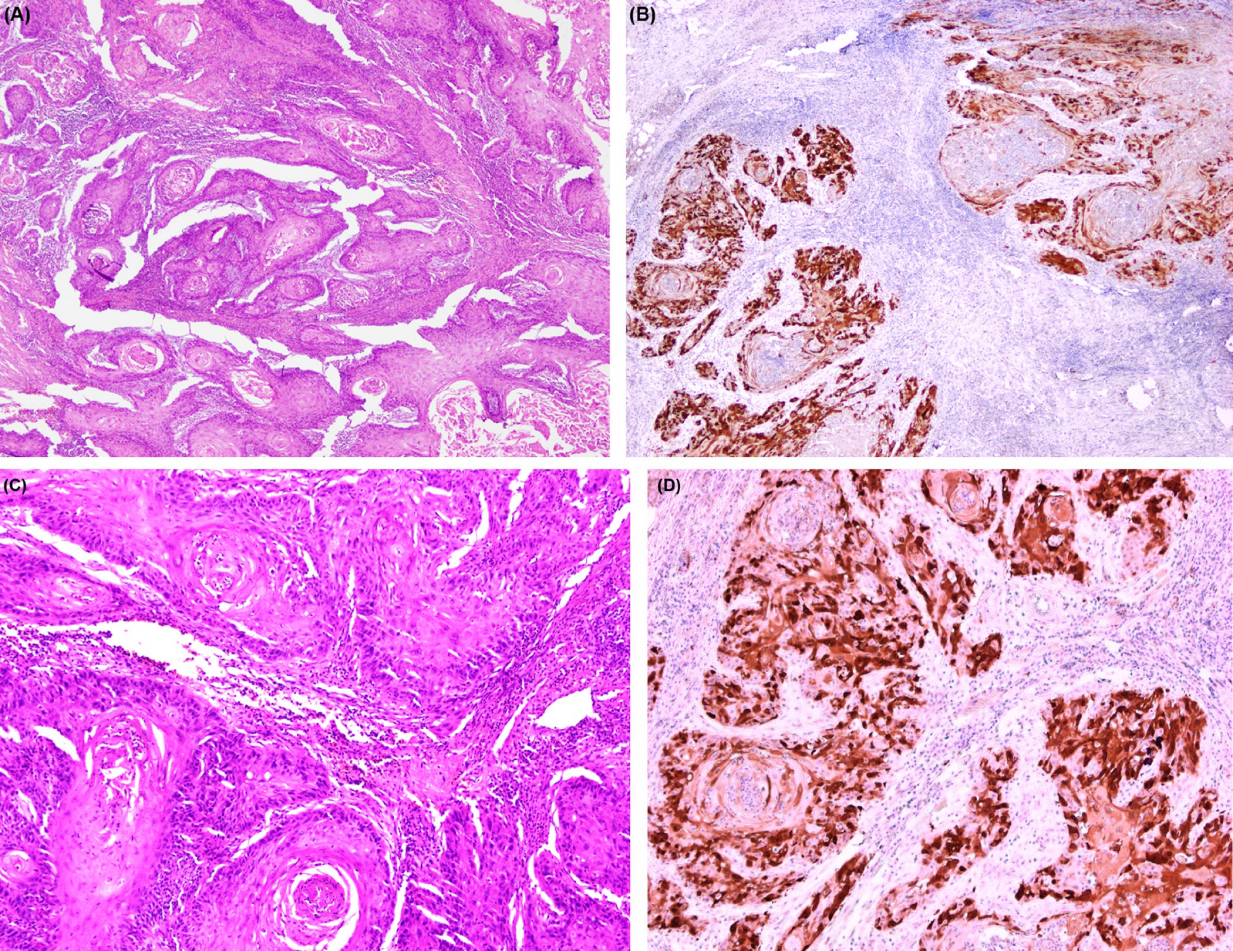
Protein expression of p16 by IHC, nuclear and cytoplasmic staining and their respective H&E staining. (A) & (B) H&E and p16 staining observed at 2X and (C)&(D) H&E and p16 staining observed at 10X.

In a subset of 50 samples of OSCC, the p16 nuclear and cytoplasmic protein expression was seen in 38% of tumours i.e., n = 19, (Fig 2A–2D). Among these 19 p16-IHC positive samples, 26% were also positive for HR-HPV (n = 5), and 73% (n = 14) were HR-HPV negative by PCR (Table 2). However, no signification was found between HR-HPV presence by PCR testing and p16 expression by IHC (p>0.09).

**Table 2:** Correlation between HR-HPV positivity by PCR and p16 protein expression by IHC.

		HR-HPV-PCR N = 50		Total	p-value χ^2^
		Negative-n (%)	*Positive-n (%)		
**p16-IHC**	**Negative**	29 (93.5)	2 (6.5)	31	0.09 ns
	* **Positive**	14 (73)	5 (26)	19	
**Total**		43	7	50	

•HR-HPV detection by PCR and p16 protein expression by IHC in a total of 50 cases.

•*HR-HPV and p16 positivity 26% (n = 5).

•p>0.05 is non-significant (ns).

Descriptive analysis was performed for HR-HPV+ve and p16+ve samples to assess the frequency of chewing overall, areca nut, betel quid, and gutka. In p16+ve samples (n = 19), 63.2% were chewers for any of the three types, and the majority had the habit of chewing betel quid and gutka. Whereas, in HR-HPV+ve (n = 7), not much difference was observed in chewing of different substances (Table 3).

**Table 3:** Descriptive analyses of positive p16- IHC and HR-HPV for chewing (overall), betel quid, areca nut, and gutka*.

Total N = 50	Chewing overall n (%)		Betel quid n (%)		Areca Nut n (%)		Gutka n (%)	
	No	Yes	No	Yes	No	Yes	No	Yes
**p16-IHC**								
Positive n = 19	7 (36.8)	12 (63.2)	10 (52.6)	9 (47.4)	17 (89.5)	2 (10.5)	12 (63.2)	7 (36.8)
**HR-HPV**								
Positive n = 7	3 (42.9)	4 (57.1)	4 (57.1)	3 (42.9)	5 (71.4)	2 (28.6)	5 (71.4)	2 (28.6)

•“n” represents the total number of positive samples for gene or protein biomarker

•*Gutka is most used in this population and is a mix of tobacco, areca nut, slaked lime, flavours, wax

HR-HPV and p16 positivity did not show any significance in univariate or multivariate regression analysis when evaluated for the association with chewing habits and substances. Despite the insignificance, p16 positive samples had 1.8 more odds of having chewing habits (95% CI: 0.56–5.88) (Table 4).

**Table 4:** Univariate regression model of age, gender, p16 and HR-HPV and chewing products, N = 50.

Variables	Chewing overall		Univariate analysis	
	Yes-n (%)	No-n (%)	OR (95% CI)	p-value
**Age (Mean±SD**)	50.1±14.5	49.3±13.9	1.00 (0.96–1.04)	0.83
**Gender**				
Female	7 (50)	7 (50)	1 (Reference)	
Male	20 (55.6)	16 (44.4)	1.25 (0.36–4.31)	0.72
**p16-IHC**				
Negative	15 (48.4)	16 (51.6)	1	
Positive	12 (63.2)	7 (36.8)	1.80 (0.56–5.88)	0.31
**HR-HPV-PCR**				
Negative	23 (53.5)	20 (46.5)	1	
Positive	4 (57.1)	3 (42.9)	1.10 (0.23–5.81)	0.86

•The model was adjusted for age and gender due to the biological importance of variables

•p<0.05 significant, OR 1 as reference.

When analyzed for association with areca nut, HR-HPV had 5.33 more odds of having areca nut chewing habits (95% CI: 0.71–40.05) (Table 5). None of the variables was significant in multivariate regression models; however, HR-HPV presented odds of 3.54 with a broad 95%CI window (0.36–34.04).

**Table 5:** Logistic regression analysis of Areca nut with gene and protein expressions as independent factors, N = 50.

Variables	Areca Nut use		Univariate analysis	
	Yes-n (%)	No-n (%)	OR (95% CI)	p-value
**Age** (Mean±SD)	59±24	48.78±12.63	1.05(0.98–1.12)	0.14*
**Gender**				
Female	3(21.4)	11(78.6)	1 (Reference)	
Male	2(5.6)	34(94.4)	0.21(0.032–1.46)	0.12*
**p16-IHC**				
Negative	3(9.7)	28(90.3)	1	
Positive	2(10.5)	17(89.5)	1.09(0.16–7.25)	0.92
**HR-HPV**				
Negative	3(7)	40(93)	1	
Positive	2(28.6)	5(71.4)	5.33(0.71–40.05)	0.10*

•p<0.05 and OR 1 as reference. As no significance was observed with any variable in multivariate regression analysis, so are not reported in the table.

•* represents variables adjusted in multivariate model.

Similar to areca nut, betel quid chewing showed no significant association with any of the variable, but high odds were observed with being male and p16 positivity [95% CI (0.41–6.07) and (0.67–7.22), respectively] (Table 6). When association was checked with gutka chewing, the majority of males (n = 13) with a mean age of 46±11.24 were found to be gutka chewers. Though no association was found between gender, HR-HPV, p16 and gutka chewing (p>0.05) yet males had high odds of being gutka chewers and developing OSCC (95% CI: 0.65–17.56). Gutka chewing had 1.7 times more odds of p16 positivity (95% CI: 0.49–5.74) (Table 7).

**Table 6:** Logistic regression model of Betel quid use as a dependent factor with gene and protein expression, N = 50.

Variables	Betel quid use		Univariate analysis	
	Yes-n (%)	No-n (%)	OR (95% CI)	p-value
**Age** (Mean±SD)	50.22±15.41	49.56±13.63	1.00 (0.96–1.05)	0.87
**Gender**				
Female	7 (50)	7 (50)	1(Reference)	
Male	20 (55.6)	16 (44.4)	1.60 (0.41–6.07)	0.49
**p16-IHC**				
Negative	15 (48.4)	16 (51.6)	1	
Positive	12 (63.2)	7 (36.8)	2.20 (0.67–7.22)	0.19
**HR-HPV-PCR**				
No	23 (53.5)	20 (46.5)	1	
Yes	4 (57.1)	3 (42.9)	1.4 (0.27–7.09)	0.68

• Significance level p<0.05 and OR 1 is taken as reference. No significance was observed with any variable in multivariate analysis; hence, it is not reported in the table. The model was adjusted for age and gender because of the biological importance of variables.

**Table 7:** Logistic regression analysis of HR-HPV and p16 with Gutka use, N = 50.

Variables	Gutka use		Univariate analysis	
	Yes-n (%)	No-n (%)	OR (95% CI)	p-value
**Age** (Mean±SD)	46±11.24	51.43±15.7	1.00 (0.93–1.02)	0.22*
**Gender**				
Female	2 (14.3)	12 (85.7)	1 (Reference)	
Male	13 (36.1)	23 (63.9)	3.40 (0.65–17.56)	0.14*
**p16-IHC**				
Negative	8 (25.8)	23 (74.2)	1	
Positive	7 (36.8)	12 (63.2)	1.70 (0.49–5.74)	0.41
**HR-HPV**				
Negative	13 (30.2)	30 (69.8)	1	
Positive	2 (28.6)	5 (71.4)	1.00 (0.16–5.39)	0.92

•*p-values were adjusted in the multivariate model along with age, -OR 1 is taken as reference, and p<0.05 as significant

## Discussion

HR-HPV prevalence in HNSCC has shown wide variation globally, possibly due to the anatomic site involved in the tumour, gender, and geographic location [17]. In HNSCC, HR-HPV has shown a strong causal relationship with OPSCC and tonsillar cancer and a much weaker association with the oral cavity. In a comprehensive SEER analysis, HPV+ve HNSCCs encompass the oropharynx, whereas HPV-ve HNSCCs involve the oral cavity [18]. In a large case-control study done in nine countries, HR-HPV was found in 3.9% of patients with oral cavity cancer across Europe, Asia, Africa, and America. The majority had no chewing/smoking habits [19]. Multicentre studies conducted in two different countries reported 8.1% [20] and <4% [21] HR-HPV prevalence in OSCC. More et al. reported a 6.6% and 20% comparative prevalence with and without tobacco history, respectively [22]. A study on 187 OSCC samples in Korea presented the HR-HPV prevalence of 4.3%, and no association was found with age, gender, tumor stage and site [23]. In Brazil, HR-HPV is reported to be 4.69% with high heterogeneity [24]. According to the College of American Pathologists (CAP) recent guidelines, the pathologist should not perform HR-HPV testing in a routine on non-OPSCC tumors. According to their systematic report, when all types of HR-HPV testing combined, the positivity ranges from 5.9% to 58.3%, depending on the anatomic site [25]. Results from our study coincide with these studies as PCR based HR-HPV DNA testing using E6 primers for types 16 and 18 resulted in 3.8% positivity (among 186 samples). And among these HR-HPV positives, no significant difference was found between chewing habits. However, contrasting results have also been published from different parts of the world. A study from India reported HPV-DNA presence in 31% of patients, the majority being chewers. Hypothetically, continuous mechanical friction caused by chewing makes the user more prone to HPV infection [26]. Conversely, another case-control study from South India did not report any HR-HPV positivity, and no association was found with betel quid chewing [27]. A systematic review of HPV prevalence in South-Central Asia resulted in the extraction of 13 studies, and almost half reported a positive association between HPV and OSCC, whereas others could not find any association. The extensive chewing habits and smoking in this region make it difficult to find a conclusive association between the two [28]. A large pooled study including 3272 controls and 1157 cases reported a positive association of HR-HPV with all anatomical sites of HNSCC; however, the strongest was found with palate cancer [29]. Similarly, a meta-analysis presented the incidence rate of HR-HPV associated OPSCC as 24.14% [30].

Anatomically, ductal epithelium of salivary glands, swollen gingival pocket, cryptal epithelium of the tonsils, border of the oral cavity, and oropharynx may act as a reservoir for HR-HPV. In the oral cavity, gingival pockets are the only site where basal cells that could act as HR-HPV infection sites are exposed [31]. Another reason for low HR-HPV prevalence in OSCC in our region could be less common unsafe sexual practices, as it is related to unsafe practices and multiple partners in other parts of the world [32–34]. Though this question was not part of the questionnaire, it was assumed from the societal culture that prevails in our country. The prevalence difference could also be due to the type of tissue and test to be done. We performed DNA-based PCR testing where confirmed human DNA does not extensively affect the estimation [35]; however, poor fixation and low yields could provide false positives. This problem was tried to be addressed by performing triplicates.

DNA-based PCR testing does not provide evidence for transcriptionally active HR-HPV, so we performed p16 IHC to increase the sensitivity and specificity. According to CAP guidelines, p16 IHC testing is recommended for OPSCC (>70% staining cutoff), but it should not be part of routine testing for non-OPSCCs because of insignificant association. Various studies have reported the p16 protein expression ranges from 10–40% in HPV+ve OSCCs [36–38]. Another study did not show any association between the two detection methods [20], which implies to the findings of this study. The results are concordant with these studies as the combined positivity rate for both techniques was 26%, and HR-HPV DNA and p16 insignificant correlation was 71%. A comprehensive meta-analysis showed the pooled p16 positivity of 28.1% for HR-HPV DNA +ve in OSCC. They concluded that the attributable fraction (AF) for either HPV mRNA or p16 was considerably lower in OSCC [39]. Further, p16 has been shown to be a non-predictor of HPV status in OSCC [40]. This poor correlation between HPV and p16 is likely because of the epigenetic modifications or mutations in CDKN2A (gene encoding p16) [41]. Another reason could be the use of conventional PCR for HPV DNA testing rather than Nested PCR or sequencing.

In our study, HR-HPV independent p16 expression was observed in 73% (n = 14/19) which infers the activation of a different mechanism. Studies have stated the weak concordance between p16 and HR-HPV positivity [42–45]. The overexpression pattern of p16 could be due to external oncogenic stimuli or tobacco-dependent tumorigenesis [46]. Deregulation of pRB (loss of heterozygosity) in the absence of HPV can also result in augmented protein expression of p16 via a positive feedback loop [47, 48]. Another proposed mechanism is the frequent presence of wild-type CDKN2A in HPV-/NSD1 mutants, suggesting a high functional p16 level irrespective of pRB mutations [49].

Moreover, our study could not find any significant association between HR-HPV and p16 with chewing habits and substance types. Studies have presented no association between HR-HPV and chewing/smoking/alcohol usage [50–52]. This suggests the importance of HR-HPV and chewing habits as independent risk factors. The absence of a significant association between p16 and chewing habits in our study is strengthened by the findings of some recent work [53]. Another study concluded that p16 had no role as a surrogate marker [54]. In a larger sample size setting, with most of the population being chewers, p16 and HR-HPV were not associated with chewing habits [55].

## Conclusion

In this study, only a handful of OSCC cases showed the presence of HR-HPV, i.e., seven out of 186 (3.8%) cases tested by PCR for HPV types 16 and 18. It was concluded that in contrast to the established significant association of HR-HPV with OPSCC (arising from the base of the tongue, palatine tonsils, soft palate, and adenoids), the OSCC association is very low in our population. Moreover, p16 could be expressed independently of HR-HPV infection by other mechanisms in OSCC. Further, no significant association with chewing habits suggests the unrelated role of HR-HPV in OSCC. However, a larger sample size could have strengthened the findings. This study could provide insight into HR-HPV independent OSCC and different molecular mechanisms alongside chewing habits being driven for this aggressive cancer.

## Supporting information

S1 Raw images (ZIP)

## Abbreviations

OC: Oral cavity
OSCC: Oral squamous cell carcinoma
HNSCC: Head & neck squamous cell carcinoma
HR-HPV: High-risk human papillomavirus
