# Supplementary figures and images for: Prevalence of high-risk human papillomavirus in oral squamous cell carcinoma with or without chewing habits
Source: PLoS One. 2024 May 1;19(5):e0300354. doi: 10.1371/journal.pone.0300354 (PMC11062528; doi:10.1371/journal.pone.0300354)

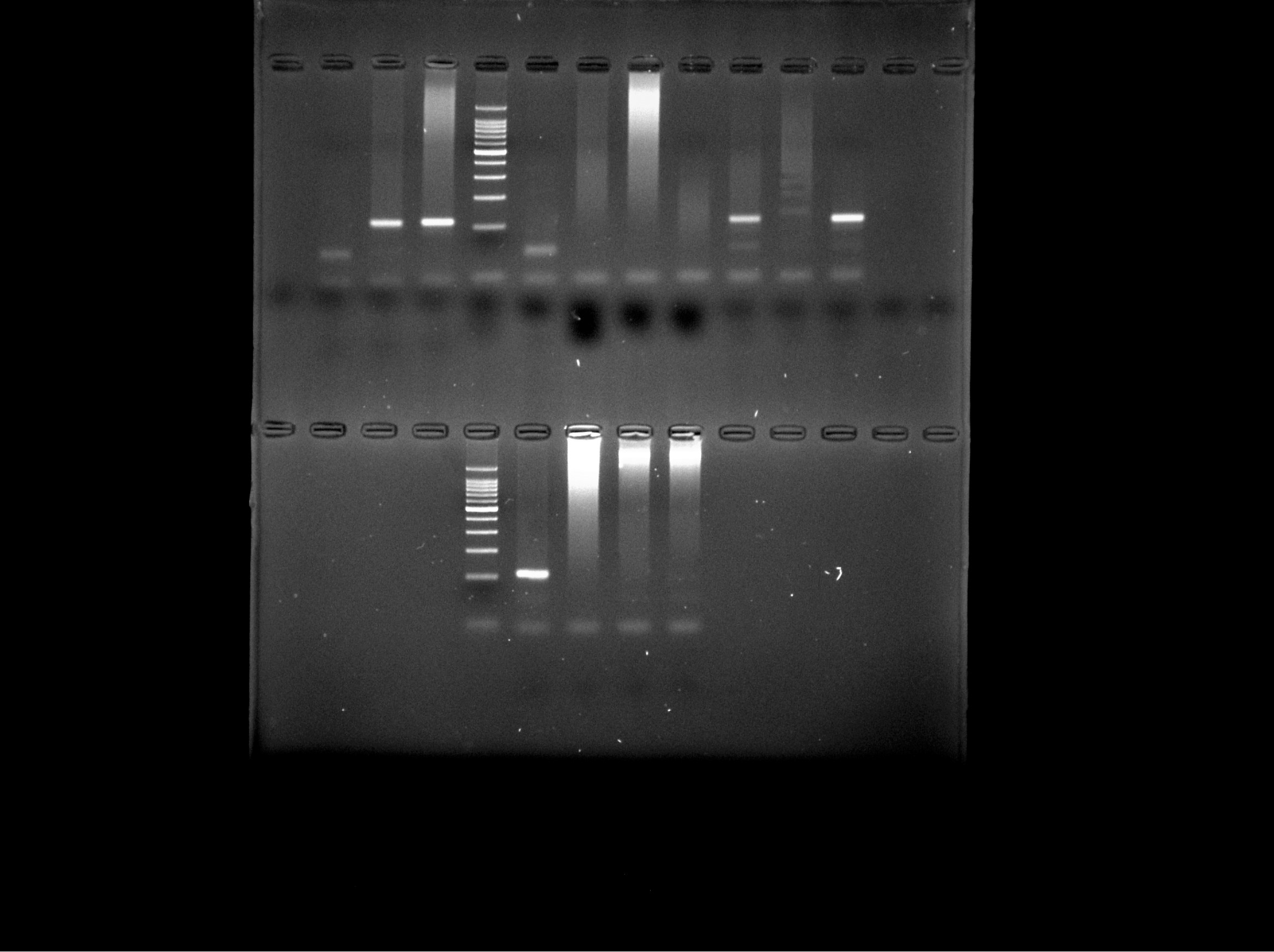

Supplement: S1 Raw images — (ZIP) [file pone.0300354.s001.zip › S1_raw_images/AKUH 2018-04-12-HPV 18.jpg]

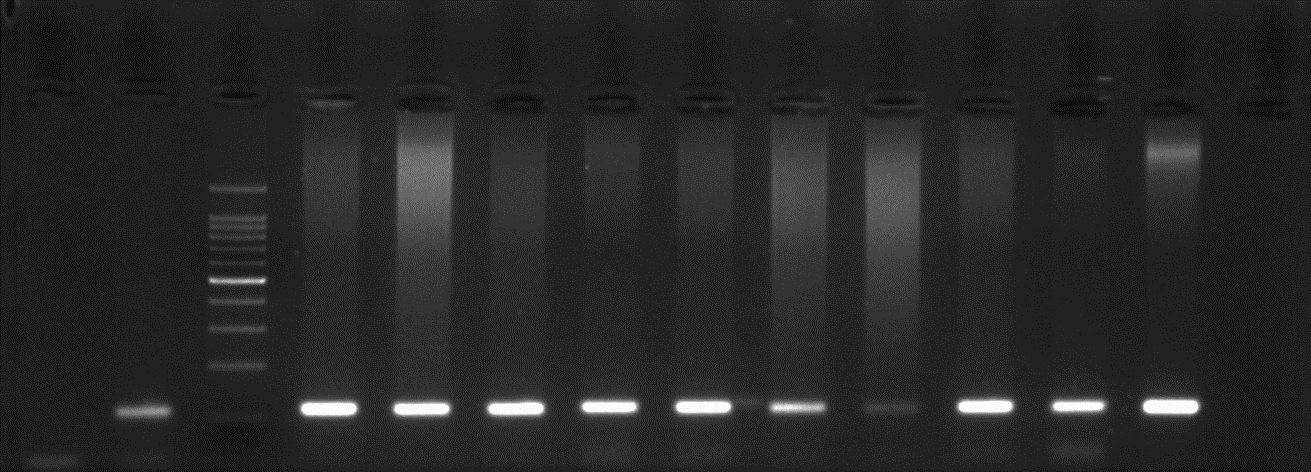

Supplement: S1 Raw images — (ZIP) [file pone.0300354.s001.zip › S1_raw_images/beta globin.png]

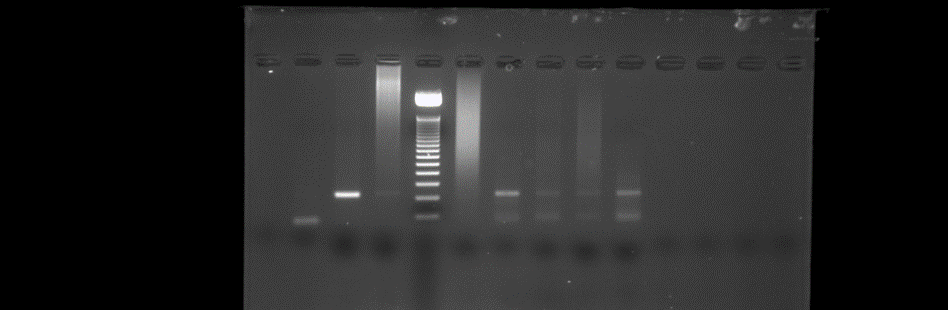

Supplement: S1 Raw images — (ZIP) [file pone.0300354.s001.zip › S1_raw_images/HPV 16 raw.png]
